# Effect of Canagliflozin on Urinary Albumin Excretion in Japanese Patients with Type 2 Diabetes Mellitus and Microalbuminuria: A Pilot Study

**DOI:** 10.1089/dia.2018.0169

**Published:** 2018-09-26

**Authors:** Takeshi Osonoi, Maki Gouda, Mamiko Kubo, Kenji Arakawa, Toshio Hashimoto, Masanori Abe

**Affiliations:** ^1^ Department of Internal Medicine, Naka Kinen Clinic, Ibaraki, Japan.; ^2^ Ikuyaku, Integrated Value Development Division, Mitsubishi Tanabe Pharma Corporation, Tokyo, Japan.; ^3^ Division of Nephrology, Hypertension and Endocrinology, Department of Internal Medicine, Nihon University School of Medicine, Tokyo, Japan.

**Keywords:** Canagliflozin, SGLT2 inhibitor, Type 2 diabetes, Albuminuria, Nephropathy, Renoprotective effect

## Abstract

***Background:*** Albuminuria characterizes the progression of kidney injury. The effect of canagliflozin on the excretion of microalbumin was assessed for investigating its renoprotective potential in Japanese patients with type 2 diabetes mellitus (T2DM).

***Patients and Methods:*** Twenty Japanese patients with T2DM and microalbuminuria were enrolled and administered with 100 mg of canagliflozin once a day for 12 weeks. These subjects were admitted to the clinic at the start and end of the treatment period for 24-h urine collection. The primary endpoint was the percentage change in geometric mean 24-h urinary albumin excretion from baseline to week 12.

***Results:*** The urinary albumin level decreased by 42.0% (95% confidence interval: 21.9–57.0; *P* = 0.0011) after 12 weeks of canagliflozin treatment. A number of blood and urinary parameters also significantly decreased, including hemoglobin A1c, fasting plasma glucose, estimated glomerular filtration rate, and creatinine clearance, while hematocrit was elevated. Among the biomarkers associated with kidney injury and inflammation, the urinary level of the oxidative stress marker 8-hydroxy-2′-deoxyguanosine was also decreased. There were no meaningful correlations noted between changes in urinary albumin excretion and other parameters/biomarkers. No severe adverse events were reported over the 12-week treatment period.

***Conclusions:*** The results of this study indicate that canagliflozin decreases microalbuminuria in Japanese patients with T2DM. Albuminuria could be reduced as a result of changes in various physiological pathways; therefore, it is imperative that future, large-scale, studies attempt to determine the detailed mechanisms involved. Canagliflozin may offer a novel therapeutic option for Japanese patients with T2DM and incipient nephropathy.

## Introduction

Diabetic nephropathy is the most common cause of end-stage kidney disease (ESKD) and the incidence and prevalence of this condition have both dramatically increased worldwide.^1,2^ Both micro- and macroalbuminuria are predictors of progressive kidney diseases, as has been demonstrated in several prospective and epidemiological studies, particularly in patients with type 2 diabetes mellitus (T2DM).^3,4^ However, despite treatment with currently recommended therapy, numerous patients with T2DM exhibit significant albuminuria and progress toward ESKD.^1,2^ Previous studies have demonstrated a racial difference in the incidence of diabetic nephropathy and ESKD, and also that Asian patients with DM, as well as African Americans, Hispanics, and Native Americans, had a higher prevalence of diabetic nephropathy compared with Caucasian patients.^5–9^ In Japan, the percentage of new dialysis patients with diabetic nephropathy as the primary disease has been reported to be 43.8%.^10^ Therefore, an additional therapeutic strategy for preventing the progression of kidney disorders in patients with T2DM is urgently required, particularly for patients of specific ethnic groups, including those of Japanese origin.

Canagliflozin is a sodium glucose cotransporter 2 (SGLT2) inhibitor that increases the urinary excretion of glucose by inhibiting urinary glucose reabsorption in the proximal tubule.^11^ Several clinical studies have demonstrated that canagliflozin can control hyperglycemia and reduce body weight and blood pressure in patients with T2DM.^12–15^ In addition, SGLT2 inhibitors are thought to exert renoprotective effects by enhanced tubuloglomerular feedback, leading to a reduction in hyperfiltration,^16–18^ and by reducing inflammatory, fibrotic, and hyperplastic responses of proximal tubular cells via blocking glucose flow into the proximal tubule cells.^19^ In a phase 3 trial, compared with glimepiride, canagliflozin reduced urinary albumin excretion and caused an initial reduction in estimated glomerular filtration rate (eGFR), followed by a stabilization of kidney function.^20^ In the Canagliflozin Cardiovascular Assessment Study (CANVAS) Program, which aimed to assess cardiovascular outcomes, canagliflozin was also noted to suppress the progression of albuminuria and reduce composite renal outcomes in patients with T2DM with high cardiovascular risk.^21^ However, Japanese patients were not enrolled in these clinical studies. In phase 3 studies targeting Japanese patients with T2DM, albuminuria was only assessed in spot urine samples.^12,13^ This particular study showed slight reductions in albuminuria. However, because albumin levels in spot urine can be influenced by numerous factors, including circadian rhythm, hydration status, and physical activity, spot levels represent a less reliable measure for assessing renal impairment.^22^ Consequently, it was not concluded whether canagliflozin exerted renoprotective effects in these patients.

In the present study, we aimed to examine the renoprotective effect of canagliflozin in Japanese patients with T2DM and microalbuminuria via 24-h urine collection. For further characterizing the effect of this treatment on kidney, we also investigated changes in biomarkers for kidney injury, inflammation, and oxidative stress.

## Patients and Methods

### Patients

Male and female patients (age 20–74 years) with T2DM exhibiting an albumin-to-creatinine ratio of 30.0–299.9 mg/g creatinine in a first-morning-void urinary test were selected. Subjects were treated using diet and exercise therapy alone, or in combination with antidiabetic drug therapy other than SGLT2 inhibitors, insulin, and glucagon-like peptide-1 receptor agonists. Other inclusion criteria were as follows: hemoglobin A1c (HbA1c) ≤10%, systolic blood pressure (SBP)/diastolic blood pressure (DBP) <140/90 mmHg, and eGFR ≥45 mL/min/1.73 m^2^. eGFR was calculated according to the Japanese eGFR equation on the basis of serum creatinine (eGFRcreat).^23^

### Study design

This study was a single-site, single-arm, open-label study aimed at evaluating the effect of canagliflozin on urinary microalbumin levels in patients with T2DM. We set our exploratory sample size at 20 subjects for feasibility of the study with an estimation accuracy of 95% confidence interval (CI), equating to ∼1/2 of the standard deviation (SD). Canagliflozin (100 mg) was administered once a day before or after breakfast for 12 weeks. Subjects were instructed not to change their diet and exercise regimen during the study and keep the dosage and administration of all concomitant medications constant. Subjects were admitted to the Naka Kinen Clinic 1 day before the start and end of the 12-week treatment period for 24-h urine collection. Spontaneous reports of adverse events (AEs) were collected from patients at the hospital visits. AEs were classified according to the Medical Dictionary for Regulatory Activities/Japanese Edition (MedDRA/J) version 20.0. This study was conducted between August 2016 and December 2017 in accordance with the Declaration of Helsinki (revised in October 2013). Written informed consent was obtained from all individuals before participation in this study. The protocol was registered in the UMIN Clinical Trial Registry as UMIN000023959 and was approved by the Institutional Ethics Review Committee of Naka Kinen Clinic. Members of the Clinical Operations Department, DOT World Co., Ltd. (Tokyo, Japan) performed monitoring and auditing in accordance with the Japanese governmental ethical guidelines and the study protocol.

### Endpoints

The primary endpoint was the percentage change in geometric mean 24-h urinary albumin excretion from baseline to week 12. Secondary endpoints included changes in laboratory data. Any AEs that occurred during the period of ongoing canagliflozin treatment were recorded.

The following parameters were measured in 24-h urine samples: albumin, transferrin, *N*-acetyl-β-d-glucosaminidase (NAG), liver-type fatty acid binding protein (L-FABP), β2 microglobulin, 8-hydroxy-2′-deoxyguanosine (8-OHdG), aldosterone, and creatinine. The following urinary parameters were determined in first-morning-void urine samples, and the results expressed as a ratio to urinary creatinine as follows: albumin, type IV collagen, neutrophil gelatinase-associated lipocalin (NGAL), kidney injury molecule-1 (KIM-1), and creatinine. Blood was collected for assessing blood biochemistry, including plasma interleukin (IL)-18, serum high-sensitivity C-reactive protein (CRP), plasma tumor necrosis factor receptor (TNFR)1 and plasma TNFR2, serum erythropoietin, serum creatinine, serum cystatin C, serum uric acid, plasma renin activity, and plasma aldosterone. All these parameters were measured at LSI Medience Corporation (Tokyo, Japan). HbA1c, fasting plasma glucose, body weight, blood pressure, 24-h water intake, and 24-h urine volume were measured at Naka Kinen Clinic. eGFR was calculated using both the Japanese eGFRcreat^23^ and the Japanese eGFR equation on the basis of serum cystatin C (eGFRcys).^24^ Creatinine clearance was determined by serum creatinine and 24-h urinary creatinine excretion.

### Statistical analyses

Data are presented as mean ± SD or mean with 95% CI. Considering the skewed distribution of data, 24-h urinary albumin and the albumin-to-creatinine-ratio in first-morning-void urine samples were log transformed for analysis. The results of the mean changes and 95% CI in the log transformed data from baseline to 12 weeks were back transformed (anti-log) to obtain geometric means of the ratios of the 12-week value to the baseline value and corresponding 95% CI. The values were then expressed as the percentage change from the respective baseline values. Changes from baseline were analyzed using two-tailed paired *t*-tests. Next, associations between the primary endpoint and other variables were assessed using Spearman's correlation coefficients. *P-*value of <0.05 was considered statistically significant. Because the statistical analyses of secondary endpoints were conducted exploratory, we did not consider multiplicity. All statistical analyses were performed with SAS software version 9.4 (SAS Institute, Inc., Cary, NC). Data management and statistical analyses were conducted by the Data Science Department, DOT World Co., Ltd.

## Results

### Patients

A total of 20 subjects (15 males and 5 females) were treated with 100 mg of canagliflozin for 12 weeks. None of the subjects withdrew after consenting to the study. At baseline, these subjects had an HbA1c level of 7.30 ± 0.91% and were 62.9 ± 8.6 years old. Mean body weight was 67.3 ± 11.1 kg, mean body mass index was 26.2 ± 4.4 kg/m^2^, and the mean duration of type 2 diabetes was 13.6 ± 9.3 years. Twelve patients (60%) were taking an angiotensin receptor blocker.

### Primary endpoint

**FIG. 1.**
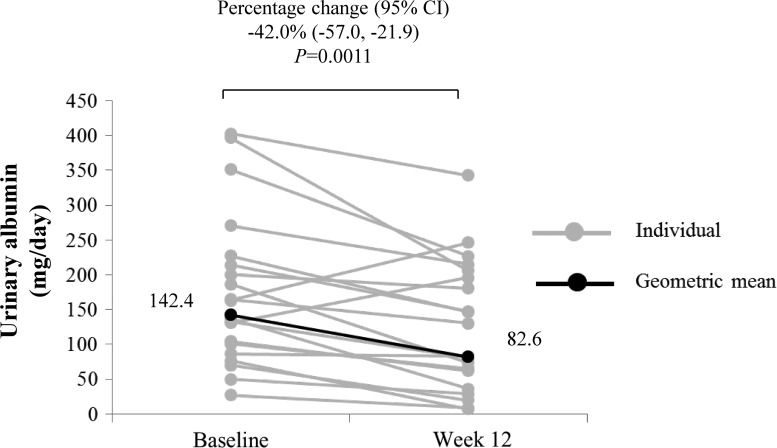
Change in 24-h urinary albumin from baseline to week 12. Data are expressed as individual measurements (gray circles) and geometric mean (black circles and values in the figure). Statistical comparison for the change in geometric mean from baseline to week 12 was conducted using a paired *t*-test. Arithmetic mean ± SD at baseline and week 12 were 174.7 ± 109.6 and 125.2 ± 93.5 mg/dL, respectively. SD, standard deviation.

As shown in Figure 1, patients had a geometric mean 24-h urinary albumin of 142.4 mg/day at baseline and 82.6 mg/day after 12 weeks of treatment with canagliflozin. The geometric mean 24-h urinary albumin significantly decreased by 42.0% (*P* = 0.0011, Fig. 1). For reference, we also assessed the effect on albumin-to-creatinine ratio using first-morning-void urine samples. The geometric mean albumin-to-creatinine ratio tended to decrease by 22.1% (*P* = 0.0876; Table 1).

**Table 1.** Change in Albumin-to-Creatinine Ratio in First-Morning-Void Urine from Baseline to Week 12

*Variable*	*Baseline*	*Week 12*	*Percentage change (95% CI)*	P *value*
Albumin-to-creatinine ratio, mg/g·Cr	86.1	67.1	−22.1 (−41.7 to 4.1)	0.0876

Data are expressed as geometric mean (95% CI) and *P-*value by paired *t*-test (*n* = 20). Arithmetic mean ± SD in albumin-to-creatinine ratio at baseline and week 12 were 104.6 ± 84.4 and 105.0 ± 99.8 mg/g·Cr, respectively.

CI, confidence interval; SD, standard deviation.

### Fasting plasma glucose, body weight, HbA1c, and blood pressure

Twelve weeks of treatment with 100 mg canagliflozin significantly reduced fasting plasma glucose and HbA1c by 24 mg/dL and 0.24%, respectively (Table 2). Body weight also significantly decreased by 1.7 kg, whereas no significant differences were detected in SBP and DBP (Table 2).

**Table 2.** Changes in Fasting Plasma Glucose, Body Weight, HbA1c, SBP, and DBP from Baseline to Week 12

*Variables*	*Baseline*	*Week 12*	*Change*	P *value*
Fasting plasma glucose, mg/dL	151 ± 22	128 ± 18	−24 ± 16 (−31 to −16)	**<0.0001**
Body weight, kg	67.3 ± 11.1	65.6 ± 11.1	−1.7 ± 1.4 (−2.4 to −1.1)	**<0.0001**
HbA1c, %	7.30 ± 0.91	7.07 ± 0.64	−0.24 ± 0.45 (−0.45 to −0.02)	**0.0318**
SBP, mmHg	134.3 ± 11.6	131.9 ± 8.9	−2.4 ± 15.6 (−9.7 to 4.9)	0.4998
DBP, mmHg	81.3 ± 9.6	80.4 ± 9.9	−0.9 ± 9.5 (−5.3 to 3.6)	0.6925

Data are expressed as mean ± SD, mean ± SD (95% CI), and *P-*value by paired *t*-test (*n* = 20). Bold texts indicate *P*-value less than 0.05.

DBP, diastolic blood pressure; HbA1c, hemoglobin A1c; SBP, systolic blood pressure.

### eGFR and creatinine clearance

eGFRcreat and eGFRcys significantly decreased by 8.9 mL/min/1.73 m^2^ and 8.0 mL/min/1.73 m^2^, respectively (Table 3). Creatinine clearance also decreased by 16.9 mL/min/1.73m^2^ (Table 3).

**Table 3.** Changes in eGFR and Creatinine Clearance from Baseline to Week 12

*Variables*	*Baseline*	*Week 12*	*Change*	P *value*
eGFRcreat, mL/min/1.73 m^2^	77.5 ± 18.1	68.6 ± 15.2	−8.9 ± 7.5 (−12.4 to −5.3)	**<0.0001**
eGFRcys, mL/min/1.73 m^2^	83.1 ± 22.8	75.2 ± 21.0	−8.0 ± 7.7 (−11.6 to −4.3)	**0.0002**
Creatinine clearance, mL/min/1.73 m^2^	107.5 ± 26.3	90.6 ± 23.3	−16.9 ± 17.0 (−24.8 to −8.9)	**0.0003**

Data are expressed as mean ± SD, mean ± SD (95% CI), and *P-*value by paired *t*-test (*n* = 20). Bold texts indicate *P*-value less than 0.05.

eGFRcreat, estimated glomerular filtration rate based on creatinine; eGFRcys, estimated glomerular filtration rate based on serum cystatin C.

### Biomarkers for tissue damage, oxidative stress, inflammation, and the renin–angiotensin–aldosterone system

Next, we investigated a range of markers for glomerular damage. While the urinary transferrin level was significantly decreased after 12 weeks of treatment with canagliflozin (Table 4), the level of urinary type IV collagen did not change significantly. Regarding urinary markers for tubular injury, L-FABP and NGAL significantly decreased and increased, respectively, after 12 weeks of canagliflozin treatment (Table 4). Urinary NAG, β2-microglobulin, and KIM-1 remained unchanged, while urinary 8-OHdG, a marker of oxidative stress, was significantly decreased after 12 weeks of treatment with canagliflozin. Levels of inflammatory markers, such as plasma IL-18 and serum high-sensitivity CRP, did not significantly change. Plasma TNFR2 levels significantly increased, while TNFR1 levels did not significantly change. Plasma aldosterone levels and renin activity were both significantly increased, while urinary aldosterone did not significantly change.

**Table 4.** Changes in Biomarkers of Tissue Damage, Oxidative Stress, Inflammation, and the Renin–Angiotensin–Aldosterone System from Baseline to Week 12

*Variables*	*Baseline*	*Week 12*	*Change*	P *value*
Urinary transferrin, mg/24 h	13.8 ± 8.9	10.8 ± 8.0	−3.0 ± 4.7 (−5.2 to −0.8)	**0.0104**
Urinary type IV collagen, μg/g·Cr	3.9 ± 2.3	3.9 ± 1.6	0.0 ± 2.0 (−1.0 to 0.9)	0.9913
Urinary NAG, U/24 h	8.4 ± 3.6	8.5 ± 3.1	0.1 ± 2.7 (−1.2 to 1.3)	0.9013
Urinary L-FABP, μg/24 h	5.3 ± 7.2	3.5 ± 5.7	−1.8 ± 2.6 (−3.0 to −0.6)	**0.0055**
Urinary β2 microglobulin, μg/24 h	145 ± 229	200 ± 517	55 ± 322 (−96 to 205)	0.4586
Urinary NGAL, ng/mg·Cr	12.3 ± 25.7	18.9 ± 37.7	6.6 ± 13.3 (0.4–12.9)	**0.0381**
Urinary KIM-1, ng/mg·Cr	1.5 ± 0.9	1.4 ± 0.7	−0.1 ± 0.7 (−0.4 to 0.2)	0.5806
Urinary 8-OHdG, μg/24 h	7.0 ± 5.1	4.6 ± 3.9	−2.4 ± 4.3 (−4.5 to −0.4)	**0.0212**
Plasma IL-18, pg/mL	313 ± 111	321 ± 106	8 ± 94 (−36 to 52)	0.7051
Serum high-sensitivity CRP, mg/L	0.9 ± 1.3	1.1 ± 1.6	0.2 ± 1.4 (−0.5 to 0.9)	0.5148
Plasma TNFR1, pg/mL	1256 ± 217	1220 ± 186	−35 ± 143 (−102 to 31)	0.2815
Plasma TNFR2, pg/mL	2587 ± 600	2826 ± 605	239 ± 502 (4–473)	**0.0468**
Plasma aldosterone, pg/mL	94.8 ± 39.2	121.4 ± 48.8	26.6 ± 43.4 (6.3–46.9)	**0.0130**
Plasma renin activity, ng/(mL·h)	2.0 ± 2.8	4.5 ± 5.8	2.5 ± 3.9 (0.7–4.3)	**0.0092**
Urinary aldosterone, μg/24 h	4.5 ± 4.1	4.7 ± 3.0	0.3 ± 2.7 (−1.0 to 1.5)	0.6669

Data are expressed as mean ± SD, mean ± SD (95% CI), and *P-*value by paired *t*-test (*n* = 20). Bold texts indicate *P*-value less than 0.05.

CRP, C-reactive protein; 8-OHdG, 8-hydroxy-2′-deoxyguanosine; IL-18, interleukin-18; KIM-1, kidney injury molecule-1; L-FABP, liver-type fatty acid binding protein; NAG, *N*-acethyl-β-d-glucosaminidase; NGAL, neutrophil gelatinase-associated lipocalin; TNFR, tumor necrosis factor receptor.

### Hematocrit, water intake, urinary volume, serum uric acid, and erythropoietin

Hematocrit significantly increased by 3.0%, whereas erythropoietin and serum uric acid levels did not significantly change (Table 5). Water intake and urinary volume significantly increased by 432 and 345 mL/day, respectively (Table 5). There was a positive correlation noted between changes in water intake and urinary volume.

**Table 5.** Changes in Hematocrit, Water Intake, Urinary Volume, Serum Uric Acid, and Erythropoietin from Baseline to Week 12

*Variables*	*Baseline*	*Week 12*	*Change*	P *value*
Hematocrit, %	41.7 ± 4.6	44.7 ± 4.7	3.0 ± 2.0 (2.0–3.9)	**<0.0001**
Water intake, mL/day	2272 ± 1145	2704 ± 1078	432 ± 693 (108–756)	**0.0118**
Urinary volume, mL/day	2759 ± 1019	3105 ± 1208	345 ± 686 (24–666)	**0.0364**
Serum uric acid, mg/dL	5.6 ± 1.4	5.4 ± 1.3	−0.2 ± 0.8 (−0.6 to 0.2)	0.2285
Erythropoietin, mIU/mL	9.8 ± 5.3	9.7 ± 5.7	−0.1 ± 3.7 (−1.8 to 1.7)	0.9245

Data are expressed as mean ± SD, mean ± SD (95% CI), and *P-*value by paired *t*-test (*n* = 20). Bold texts indicate *P*-value less than 0.05.

### Correlation between changes in 24-h urinary albumin and other parameters

The change in 24-h urinary albumin was significantly correlated with those in urinary transferrin and plasma renin activity (*r* = 0.651; *P* = 0.0019 and *r* = −0.468; *P* = 0.0374, respectively, Table 6). There were no other significant correlations between the change in 24-h urinary albumin and changes in other parameters, including HbA1c, fasting plasma glucose, body weight, and eGFR.

**Table 6.** Correlation Analysis Between Changes in 24-Hour Urinary Albumin and Other Variables

*Variables*	*Spearman's correlation coefficient*	P *values*
Urinary transferrin	0.651	**0.0019**
Plasma renin activity	−0.468	**0.0374**
Urinary L-FABP	0.423	0.0628
Urinary NAG	0.422	0.0637
SBP	0.392	0.0875
Serum uric acid	−0.331	0.1536
Plasma aldosterone	−0.330	0.1551
HbA1c	0.326	0.1610
DBP	0.323	0.1644
eGFRcys	−0.315	0.1756
Serum high-sensitivity CRP	−0.290	0.2156
Urinary aldosterone	−0.266	0.2577
Urinary β2 microglobulin	0.263	0.2619
Plasma IL-18	−0.230	0.3291
Hematocrit	0.217	0.3588
Fasting plasma glucose	−0.184	0.4364
Creatinine clearance	0.161	0.4976
Plasma TNFR2	0.134	0.5738
Erythropoietin	0.126	0.5952
Water intake	−0.120	0.6134
Urinary volume	0.078	0.7431
Body mass index	0.067	0.7782
Body weight	0.065	0.7865
Urinary type IV collagen	−0.041	0.8649
Urinary NGAL	−0.041	0.8650
eGFRcreat	0.032	0.8922
Plasma TNFR1	0.032	0.8923
Urinary KIM-1	−0.026	0.9148
Urinary 8-OHdG	−0.002	0.9950

*n* = 20. Bold texts indicate *P*-value less than 0.05.

### Safety

Over the 12 weeks of canagliflozin treatment, there were no reports of serious AEs. There was one event involving upper respiratory infection in one subject and one event of muscle pain in another subject; both these patients recovered during the treatment period. In addition, there were no notable changes in laboratory test values associated with the safety of subjects.

## Discussion

The development of albuminuria not only characterizes the progression of kidney injury^3,4^ but it can also predict cardiovascular risk in patients with T2DM.^25^ Therefore, the guidelines recommended the regular assessment of albuminuria and reducing albuminuria in patients with T2DM.^26,27^ However, many patients with T2DM worldwide, including Japan, have substantial residual albuminuria and continue to progress toward ESKD. Thus, additional treatment complementing the existing therapies remains an important unmet medical need. The present pilot study showed that 12 weeks of treatment with canagliflozin significantly reduced 24-h urinary albumin excretion in Japanese patients with T2DM and microalbuminuria.

In the present study, canagliflozin reduced 24-h urinary albumin excretion by 42%. A similar magnitude of reduction in albuminuria was previously reported following canagliflozin treatment in Western patients with T2DM.^20^ Furthermore, consistent with the results of previous non-Japanese studies,^28–30^ the changes in HbA1c, fasting plasma glucose, blood pressure, and body weight did not correlate with the albuminuria response during canagliflozin treatment. Thus, canagliflozin appears to reduce urinary albumin excretion in patients with T2DM without racial differences.

We also observed that the levels of urinary transferrin were reduced after 12 weeks of treatment with canagliflozin. Transferrin is a biomarker for glomerular damage. Furthermore, it is well known that urinary transferrin showed a high correlation with urinary albumin.^31^ Taken together, the amelioration of damaged glomerular function in these patients is suggested. In addition to glomerular damage, recent studies have also shown the involvement of tubular damage during the pathogenesis of diabetic nephropathy.^32,33^ However, the tubular damage markers evaluated in this study were all within the reference ranges both at baseline and at week 12. There was a small decrease in urinary L-FABP level, which reflected the mitigation of proximal tubular stress presumably via reducing the amount of work required following canagliflozin treatment.^34–36^ Even though this could increase albumin reabsorption, it is unlikely that increased tubular albumin reabsorption plays a major role in the reduced level of urinary albumin excretion.

In this study, eGFR was lower following 12 weeks of canagliflozin treatment. Previous investigations on SGLT2 inhibitors, including canagliflozin, putatively by activated tubuloglomerular feedback,^37^ showed an acute decline in eGFR followed by a period of maintained kidney function.^20,29,38,39^ A similar reduction in eGFR was reported with renin–angiotensin–aldosterone system inhibitors,^40–42^ and this would suggest that an intraglomerular hemodynamic effect, including the alleviation of glomerular hypertension, contributes to the reduction of albuminuria.^43,44^ In addition, the activation of inflammatory pathways and oxidative stress has been shown in the kidneys of patients with diabetic nephropathy,^45,46^ and is estimated to be a factor that initiates and sustains disease progression. However, the inflammatory biomarkers measured in this study were not suppressed and circulating TNFR2 levels slightly increased after canagliflozin treatment. Intriguingly, urinary levels of the oxidative stress marker 8-OHdG were reduced after treatment with canagliflozin. Moreover, hematocrit was elevated after treatment with canagliflozin, which is expected to deliver more oxygen to tissues to reduce ischemic stress.^34,35^ However, we did not observe any association between changes in 24-h urinary albumin excretion and either of the abovementioned variables, including eGFR, 8-OHdG, and hematocrit. Although the change in plasma renin activity was significantly correlated with change in urinary albumin, we could not conclude that it was a real finding, because the correlation was driven almost exclusively by a single subject with a very large decrease in 24-h urinary albumin and no clear trend was observed in the rest of the subjects. As the present study cannot provide conclusive evidence for the precise mechanism involved, we must assume that multiple characteristics of the drug could be involved in reducing albuminuria.

There were two AEs, and no serious AEs, reported during the treatment period. In a previous study in patients with T2DM and stage 3 chronic kidney disease (eGFR: 30–59 mL/min/1.73 m^2^), genital mycotic infections and volume depletion-related AEs were frequently observed in the canagliflozin-treated group (number of patients/number of participants [event rate]: 51/703 [7.3%] and 48/703 [6.8%], respectively) than placebo group (6/382 [1.6%] and 10/382 [2.6%], respectively),^47^ and some reports have warned that SGLT2 inhibitors could induce acute kidney injury.^48,49^ However, no volume depletion- or renal-related AEs were reported in our present short-term study, and no novel safety concerns were raised.

There are some limitations to this study that should be taken into consideration. The study was performed in a single-site, single-arm, and open-label manner, sample size was relatively small, and the study follow-up period was only 12 weeks. The results generated by the present study, therefore, warrant randomized-controlled, multicenter trials with a sufficiently large number of participants and a longer treatment period for fully confirming the renoprotective effects and for delineating the mechanism of action. Definitive evidence of the renoprotective effects of canagliflozin is expected to be provided by the following ongoing studies: Canagliflozin and Renal Endpoints in Diabetes with Established Nephropathy Clinical Evaluation Study (CREDENCE; ClinicalTrials.gov number, NCT02065791) and Effect of Canagliflozin in Type 2 Diabetic Patients with Microalbuminuria in Japanese Population (CANPIONE; UMIN trial ID: UMIN000029905).

## Conclusions

The findings of this pilot study revealed that canagliflozin significantly decreases microalbuminuria after 12 weeks of treatment in Japanese patients with T2DM. The primary mechanism for the reduction of albuminuria was not elucidated but appears to be mediated by multiple mechanisms. Canagliflozin and other SGLT2 inhibitors may offer a novel therapeutic option for Japanese patients with T2DM and incipient nephropathy.
